# Expression of Concern: PHYTOCHROME C regulation of photoperiodic flowering via *PHOTOPERIOD1* is mediated by *EARLY FLOWERING 3* in *Brachypodium distachyon*

**DOI:** 10.1371/journal.pgen.1010955

**Published:** 2023-09-19

**Authors:** The PLOS Genetics Editors

Following the publication of this article [1], concerns were raised regarding results presented in Fig 2. Specifically, the corresponding author informed PLOS that the underlying expression data reported in this study have been inappropriately manipulated.

The University of California, Davis confirmed that author DPW admitted to manipulation of the data underlying the results presented in Fig 2.

The authors are working with PLOS to try and address these issues. Meanwhile, the *PLOS Genetics* Editors issue this Expression of Concern to notify readers of the above issues.
