# Supplementary figures and images for: PHYTOCHROME C regulation of photoperiodic flowering via PHOTOPERIOD1 is mediated by EARLY FLOWERING 3 in Brachypodium distachyon
Source: PLoS Genet. 2023 May 10;19(5):e1010706. doi: 10.1371/journal.pgen.1010706 (PMC10171608; doi:10.1371/journal.pgen.1010706)

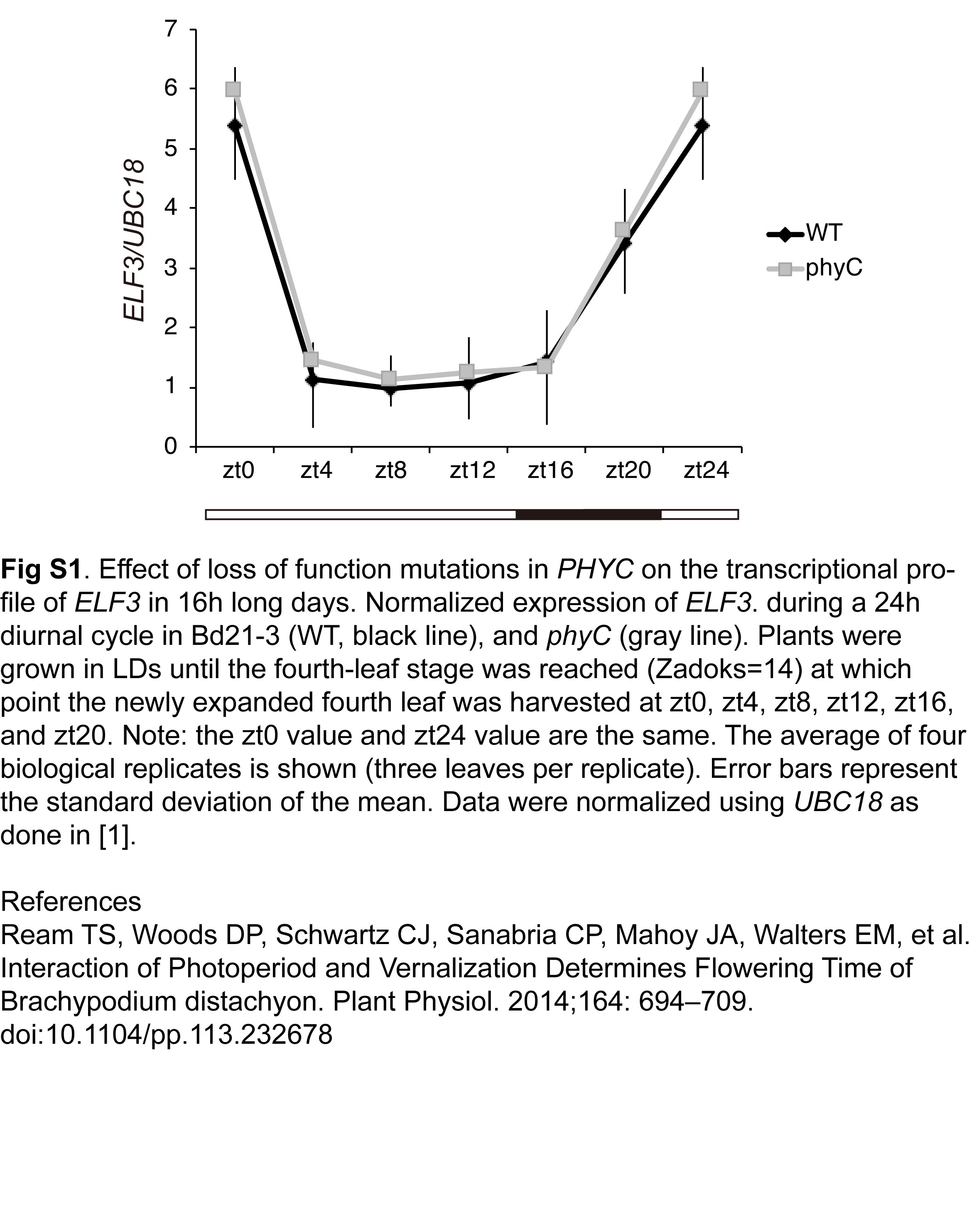

Supplement: S1 Fig — (TIF) [file pgen.1010706.s001.tif]

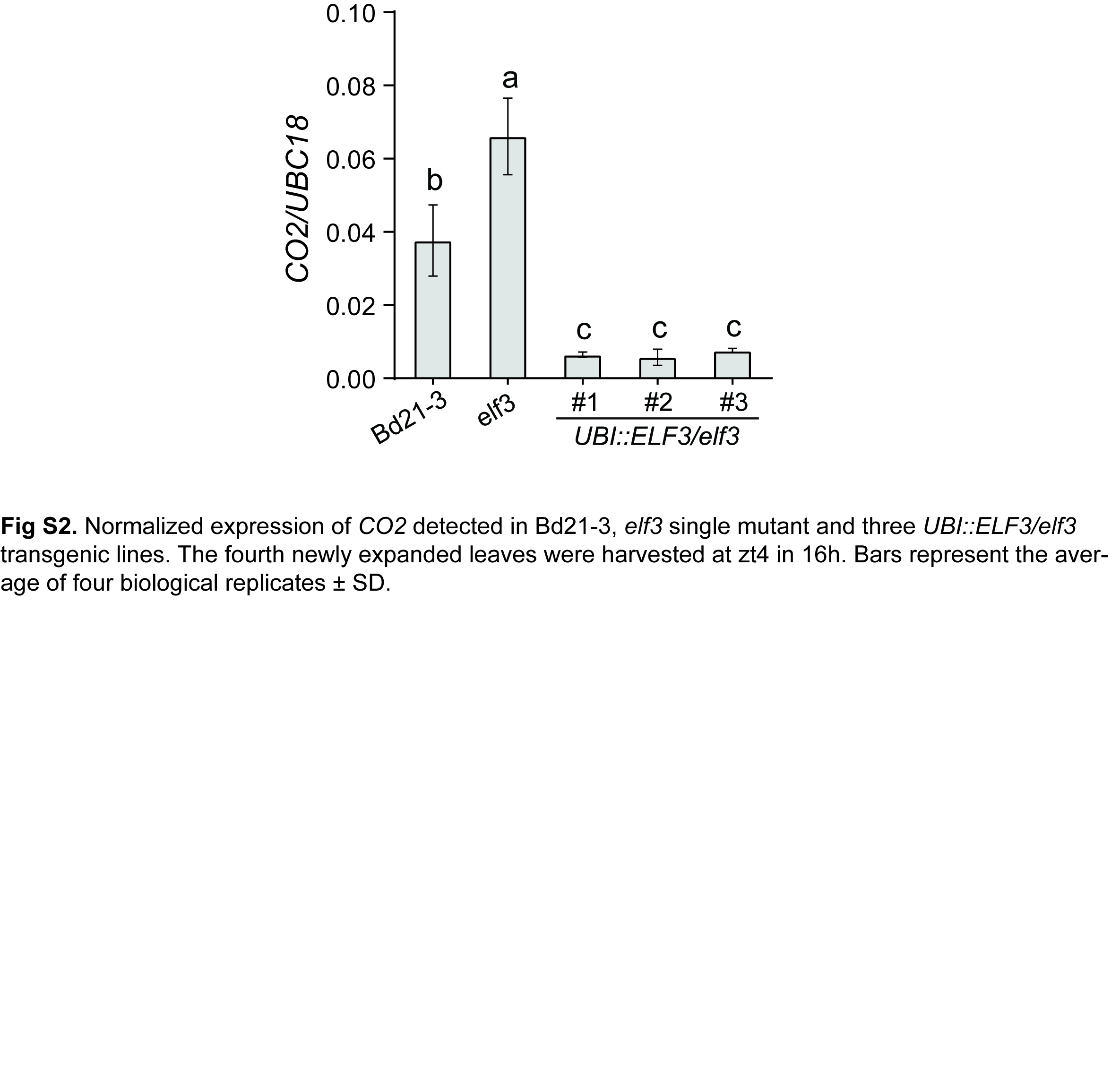

Supplement: S2 Fig — (TIF) [file pgen.1010706.s002.tif]
