# Supplementary material for: PHYTOCHROME C regulation of photoperiodic flowering via PHOTOPERIOD1 is mediated by EARLY FLOWERING 3 in Brachypodium distachyon
Source: PLoS Genet. 2023 May 10;19(5):e1010706. doi: 10.1371/journal.pgen.1010706 (PMC10171608; doi:10.1371/journal.pgen.1010706)
Supplement: S1 Table — (PDF) [file pgen.1010706.s003.pdf]

Table S1 Primers used in this study

Purpose	Primers	Sequence	NEB enzyme	Reference
qPCR	qUBC18-F	GTCACCCGCAATGACTGTAAGTTC		[1] Ream et al., 2014
	qUBC18-R	TTGTCTTGCGGACGTTGCTTTG		
	qFT1-F	TTCGGGAACAGGAACGTGTCCAAC		
	qFT1-R	AGCATCTGGGTCTACCATCACGAG		
	qVRN1-F	GCTCTGCAGAAGGAACTTGTGG		
	qVRN1-R	CTAGTTTGCGGGTGTGTTTGCTC		
	qVRN2-F	ATGCATGAGAGAGAGGCGAAGG		
	qVRN2-R	TCGTAGCGGATCTGCTTCTCGTAG		
	qPPD1-F2	CTATGCCGTCGCTTGAGTTG		This paper
	qPPD1-R2	TGCCGCCTTGATTGGAAACC		
	qCO1-F	AGAGTGGTTATGGGCTTGGA		
	qCO1-R	CTATCACCGTATTGTCTGGG		
	qCO2-F	GGCAAGTGAGGAACAGGAAAG		
	qCO2-R	TAGGCTCCACTGGTTGTTAGG		
Fine Mapping and genotyping	dCaps0241800-F	CCGTCTCCAGATTATACCTATCCG	Hpy188I	This paper
	dCaps0241800-R	GAGTGTGATTTCAGCCCTTG		
	pdd1_F	CAACCGGAGATGGTGGAAATG	Hpy166II	
	pdd1_R	GGACACTGATATTATGTTGTGC		
	dCaps0229500-F	AAAGTCCTGCTGGCGCTCGTG	BsrDI	
	dCaps0229500-R	TGACGCCGTGCTCCGGCTCTGCAA		
	elf3-F	CACCCATGCCTCCAATGTACTTCCC	Hpy166II	[2] Bouche et al., 2022
	elf3-R	GGTGGTTTCAGCTTCTGCAGGTGAA		

Reference

1) Ream TS, Woods DP, Schwartz CJ, Sanabria CP, Mahoy JA, Walters EM, et al. Interaction of Photoperiod and Vernalization Determines Flowering Time of *Brachypodium distachyon*. Plant Physiol. 2014;164: 694–709. doi:10.1104/pp.113.232678

2) Bouché F, Woods DP, Linden J, Li W, Mayer KS, Amasino RM, et al. EARLY FLOWERING 3 and Photoperiod Sensing in *Brachypodium distachyon*. Front Plant Sci. 2021;12: 769194. doi:10.3389/fpls.2021.769194
