# Supplementary material for: PHYTOCHROME C regulation of photoperiodic flowering via PHOTOPERIOD1 is mediated by EARLY FLOWERING 3 in Brachypodium distachyon
Source: PLoS Genet. 2023 May 10;19(5):e1010706. doi: 10.1371/journal.pgen.1010706 (PMC10171608; doi:10.1371/journal.pgen.1010706)
Supplement: Attachment — Submitted filename: ELF3_PHYC_PPD1_final_ response to reviewers.docx [file pgen.1010706.s005.docx]

Dear Editor,

Thank you for further consideration of our manuscript entitled “PHYTOCHROME C regulation of *PHOTOPERIOD1* is mediated by *EARLY FLOWERING 3* in *Brachypodium distachyon*” (PGENETICS-D-22-01183) for publication. We also thank the reviewers for their impressively thorough reading of our manuscript and thoughtful and helpful comments that will make the paper clearer for readers. Below we outline, point-by-point, how we have addressed the comments.

Best Regards,

Daniel Woods and Richard Amasino

**Reviewer #1**:

*This manuscript examined the genetic relationships between PHYC, ELF3 and PPD in their role in determining flowering time in B. distachyon. Previous work in B. distachyon showed a PHYC-ELF3 interaction and a reduction in ELF3 protein abundance under light irradiation in B. distachyon. The work in this paper examines the flowering time phenotypes of elf3, phyc, and elf3phyc mutants to establish that the rapid flowering of the elf3 mutant is epistatic to the delayed flowering of the phyC mutant.*

*Gene expression of select flowering time related genes are examined in these same backgrounds, with the conclusion that the transcriptional profiles of FT1, VRN1, VRN2, CO1, and CO2 are similar between elf3 and elf3/phyC mutants consistent with ELF3 acting downstream from PHYC in the photoperiod flowering pathway. A novel ppd mutant was identified and analyzed molecularly, and concluded to be a splice site mutation, with uncertain effects on truncated protein abundance, but likely a null based on comparison to a wheat ppd mutant.*

*Further experiments with this mutant followed the template used for phyC: flowering time phenotypes of ppd, elf3 and ppd elf3 double mutant descriptions. The ppd mutant is largely epistatic to elf3 with respect to flowering time, but there is more to the story that is unclear. Even more so with respect to the transcriptional profiles of FT1, VRN1, VRN2, CO1, and CO2, which varied with the gene of interest. ppd was epistatic to elf3 with respect to FT levels, but other results indicated complex interactions between PPD1 and ELF3 in the regulation of CO1 and CO2.*

*Reference to a companion paper was made, but was not available to this reviewer.*

**RESPONSE:**

The wheat paper is now available in bioRxiv: <https://doi.org/10.1101/2022.10.11.511815>

Alvarez M.A., C. Li, H. Lin, A. Joe, M. Padilla, D.P. Woods, J. Dubcovsky. EARLY FLOWERING 3 interactions with PHYTOCHROME B and PHOTOPERIOD1 are critical for the photoperiodic regulation of wheat heading time. bioRxiv doi: 10.1101/2022.10.11.511815

*1) Overall the results are largely supportive of the conclusions the authors wish to draw, but lack any type of molecular analysis that would confirm the conclusions drawn from genetics alone. Are ELF3 protein levels dependent on PHYC levels? This should be tested in their phyC mutant. ELF3 transcript levels could also be affected by the phyC mutant, and transcript levels should be tested as well. The authors reference a bioRxiv manuscript which, in many ways, goes beyond this paper, in providing evidence for a PHYC/ELF3 complex at gene promoters. However, the above suggestions of the potential of ELF3 levels (protein and/or transcript) regulated by PHYC would greatly strengthen this work.*

**RESPONSE:**

We agree, testing ELF3 protein levels in a *phy* mutant is an important experiment. Unfortunately, we have not been able to observe a sufficiently strong ELF3 protein signal using the currently available ELF3 antibody in either *B. distachyon* or in wheat. Note: the antibody to the native protein should work the same between *B. distachyon* and wheat because the peptide sequence from which the antibody is derived is identical between these two species. The lack of a signal with this ELF3 antibody is the reason why the ELF3 protein work in the companion wheat manuscript used transgenics containing ELF3 with an HA epitope tag. This work showed that the protein accumulates during the night but is degraded in the light. Thus, future plans include transgenically introducing an epitope-tagged ELF3 construct into a *phy* mutant background in *B. distachyon*, but it will require at least 8 months to generate this material.

We now include the transcriptional profile of *ELF3* in wild type and *phyC* across a diurnal cycle (see below). Similar to what has been shown in Arabidopsis and in wheat in the companion manuscript, *ELF3* mRNA levels are not altered in a *phyC* mutant background. This data is now included as a supplemental figure, see below. In the Results section we added “To determine if *PHYC* affects the expression of *ELF3*, we analyzed *ELF3* mRNA levels across a diurnal light cycle (16h light and 8h dark). There were no significant differences in *ELF3* expression at any time point in the phyC mutant relative to wildtype (Fig S1) indicating that *PHYC* does not affect the transcriptional profile of *ELF3* in *B. distachyon* consistent with results from *A. thaliana* and wheat (68, 69).”

In the discussion we now state “In *A. thaliana*, *B. distachyon*, and wheat, *phyB/phyC* mutants do not impact the circadian oscillation of *ELF3* mRNA levels [29,59,69]”

*2) Why is the peak time of CO2 in the elf3 mutant so different between Figure 2F and Figure 5F? This is the same time course with the same mutant background so results should not be so divergent. Something needs to be repeated or explained, as swapping the elf3 data between the two figures (each coming from 3 biological reps?) would lead to different conclusions.*

**RESPONSE:**

There are two things to note with respect to this variability:

First, the expression data in Fig 2 and Fig 5 were generated in different growth chambers with different light spectra (fluorescent lamps versus a combination of sodium and metal halide lamps), and *CO2* expression might be affected by the spectral differences. The difference in growth conditions is now clearly noted in the paper in the Materials and Methods "For figures 1 and 2 plants were grown at the University of California-Davis in growth chambers with metal halide and sodium bulbs as the light source and a temperature of 22 ̊C during light periods and 17 ̊C during dark periods. Light intensity was approximately 300umol m-2s-1 at plant height. For figures 4-5 plants were grown at the University of Wisconsin-Madison in growth chambers with T5 fluorescent bulbs (5,000 K), and light intensity was approximately 200umol m-2s-1 at plant height. Temperatures averaged 22 ̊C during light periods and 18 ̊C during dark periods."

Second, *CO2* is a low abundance RNA under all conditions we have examined, and the extent of differences was perhaps exaggerated by our use of an expanded y-axis. In the revised version, we have adjusted the y-axis to be similar to *CO1* in the figures, see below. We also note the only points we make from these data is that when all the genotypes are grown under identical conditions, i) *CO2* expression in the *elf3/phyC* is more similar to *elf3* which is consistent with *ELF3* acting downstream of *PHYC* in the photoperiodic flowering pathway and ii) whereas *CO2* expression is more complex in the *elf3/ppd1* mutant, *CO2* expression is similar to *ppd1* single mutants during the morning but was more similar in *elf3* single mutants during the evening. In results we state "In the *elf3/ppd1* mutant plants, *CO1* and *CO2* expression were more similar to *ppd1* in the morning and to *elf3* in the evening (Fig 5C, F). These results indicate complex interactions between *PPD1* and *ELF3* in the regulation of *CO1* and *CO2*."

Fig 2	Fig 5

*3) Also, error bars are massive for A and D in Figure 5 for elf3.*

**RESPONSE:**

One reason is that we chose a developmental stage (newly expanded fourth leaf) in which plants of all the different genetic backgrounds grown in 16h daylengths would be at an equivalent developmental stage including at the meristem level. Given the very rapid flowering of the *elf3* mutant, this can be a bit tricky to accomplish. At the fourth leaf stage, *elf3* plants are at the cusp of transitioning. At this stage, there is more variability in the induction of genes that promote flowering such as *FT1* and *VRN1* leading to large error bars. We also chose this developmental stage because we wanted to examine the expression of more genes in addition to *FT1* and *VRN1* and this stage covers more ground. In the Results section on page 12 we now include “The newly expanded fourth leaf was harvested for gene expression analyses because at this developmental stage in 16h daylengths meristems of all of the plant genotypes are at a vegetative stage and thus are developmentally equivalent.” We hope this provides some clarity as to why we chose this developmental stage for expression work.

A second reason for more observed variation in Fig 5 as compared to Fig 2 is because in Fig 2 there is one more replicate and also two more leaves per replicate.

*4) P. 20: “….elf3 mutant compared with wild-type however from zt 12-20 expression levels were lower than wild-type with a profile similar to elf3/ppd1 mutant plants.” Based on stats elf3 and WT are not different for CO2 between ZT 12-20, just CO1.*

**RESPONSE:**

Thank you for pointing this out. We changed the sentence to more accurately reflect the statistically relevant expression differences. Pg 20 in Results now reads. “However, at zt16 and zt20 expression levels were similar in wild-type, *elf3*, and *elf3/ppd1* mutants, whereas at zt12 the expression of *CO1* was reduced in *elf3* compared to wild type.”

*5) Figure 4H shows 3 lines not flowering (heading) at all but 4I indicates number of leaves at time of heading in two of those lines [ppd1 and elf3ppd1[]. How can that be? Clarify. Also in legend, must mean:*

*Flowering times under 20h (B), 16h (E), 8h (H) measured as days to heading of Bd21-3,*

*elf3, ppd1, and elf3/ppd1.*

**RESPONSE:**

In the experiment reported in our first submission, wild type and *ppd1* plants failed to head. Wild type continued to produce new leaves indicating the meristem was still vegetative; however, *ppd1* plants stopped producing leaves suggesting that the meristem had transitioned to flower formation but had not yet elongated to head (a behavior that occurs in grasses). We also found it surprising that *ppd1* transitions to flowering earlier than wild type in short days whereas it was much later to flower in long days. This prompted us to repeat the experiment at both UW Madison and UC Davis; these experiments were ongoing at the time of submission of this manuscript. In these replicated experiments, both wild type and *ppd1* plants fail to head or transition to flower based on continual growth of new leaves (shown in the figure below). Given these two subsequent replicated experiments are consistent with one another, we hypothesize that the cessation of leaf production in *ppd1* in the first experiment was an anomaly (due perhaps to a stress to which *ppd1* is particularly susceptible) and believe that the more recent data obtained at both UW Madison and UC Davis more accurately reflects the flowering phenotype of the *ppd1* mutant grown in short days. Accordingly, we have updated panel 4I as shown below, and in Results we now state:

“Under all photoperiods the *elf3/ppd1* double mutant plants flowered significantly later than the *elf3* single mutant plants (Fig 4). Interestingly, under 20h LD *elf3/ppd1* double mutant plants flowered earlier than ppd1 by 16.2 days and forming 3 fewer leaves whereas under 16h days *elf3/ppd1* mutant plants flowered later than ppd1 by 13.7 days with 2.5 more leaves. In 8h SD, only *elf3* mutant plants were able to flower whereas Bd21-3, *ppd1*, and *elf3/ppd1* all failed to flower by the end of the experiment. It is also worth noting that *elf3/ppd1* double mutant plants are still able to respond to different photoperiods, with longer days resulting in significantly earlier flowering plants than under shorter days (Fig 4B, E, H). Taken together, these results indicate that there are strong genetic interactions between *ELF3* and *PPD1* under different photoperiods, that *PPD1* is a key downstream gene of *ELF3*, and that there is a residual photoperiodic response that is independent of these two genes.”

In order to be fully transparent about the flowering of *ppd1* in SD we added the following details to the legend for Fig. 4.

“The 8h experiment was repeated three times. The first experiment resulted in *ppd1* plants that stopped producing new leaves before wild-type. One possibility for the cessation of new leaf production in *ppd1* plants in this experiment is that the meristem transitioned to flowering but then did not proceed to heading. However, in two subsequent experiments *ppd1* plants continually produced new leaves for the duration of the experiment similar to wild type and this data is shown in (I). Data for all three experiments are shown in supplemental data for Fig. 4. When grown under non-inductive conditions for 120 days or more, a few *B. distachyon* plants can flower; we consider this a stochastic flowering response because the majority of plants do not flower.”

We also removed the portion of the Discussion that involved the flowering of *ppd1* in 8h SD.

*6) On page 21; Fig 6B should be Fig 6C. Fig 6C and F should be Fig 6D and G.*

**RESPONSE:**

Thank you for catching this error; it is corrected.

*7) On page 24; Fig 6 should be Fig 6A and B.*

**RESPONSE:**

Corrected

*8) On page 25; Fig 6 should be Fig 6A and B.*

**RESPONSE:**

Corrected

**Reviewer #2:**

*The authors provide a concise genetic analysis of photoperiodic flowering in the temperate grass Brachypodium. Through the phenotypic and molecular analysis of single and double mutants they show that ELF3 acts downstream of PHYC in the induction of the flowering activator PPD1, with ELF3 being a negative regulator of PPD1 expression. For this study, the authors identified a ppd1 mutant which was not available before.*

*The authors´conclusions are supported by the data. The manuscript is well-written. I only have a few minor comments:*

*1) Fig. 4H,I. Fig. 4H shows that the mutants ppd1 and elf3/ppd1 fail to head within the time frame of the experiment. Nevertheless, the authors show in Fig. 4H the number of leaves at the time of heading. That seems to be a discrepancy to me. Can you please explain?*

**RESPONSE:**

This issue was also raised by Reviewer 1, question 5. Please see our response to question 5 from Reviewer 1.

*2) Fig. 5A, D: The transcript levels of FT1 and VRN1 cannot be seen in the WT and ppd1 mutants. I assume that they are not seen because they are as low as in the elf3/ppd1 mutant. Can you please indicate that or make them visible in another way? On the other hand, would it not be expected that FT1 and VRN1 transcript levels in the WT are as high as in the elf3 mutant in LD because these genotypes flower at the same time in LD (see Fig. 4a)?*

**RESPONSE:**

Thank you for the suggestion. We have now added the expression values for *FT1* and *VRN1*, see figure image below, and the raw data is also now included in supplemental data, which we indicate in the figure legend. We have added the following sentence to the figure legend to add clarity to the presentation of the data: “The black, gray, and orange lines overlap given the scale used to show *ELF3* expression in the same graph; the orange line is arbitrarily shown on top.”

Fig 4A shows heading in 20h LD but the expression work was done in 16h LD (Fig 4D). In 16h LD, there is a difference in heading between *elf3* and WT, with *elf3* flowering more rapidly. The expression work in Fig 5 was done in 16h and is stated in the figure legend “Effect of loss of function mutations in *ELF3*, and *PPD1* on the transcriptional profiles of six flowering time genes in 16h long days.” Thus, one would expect to see differences in expression for genes such as *FT1* and *VRN1*.

**Have all data underlying the figures and results presented in the manuscript been provided?**

Large-scale datasets should be made available via a public repository as described in the *PLOS Genetics* [data availability policy](http://journals.plos.org/plosgenetics/s/data-availability), and numerical data that underlies graphs or summary statistics should be provided in spreadsheet form as supporting information.

Reviewer #1: Yes

Reviewer #2: None

**RESPONSE:**

An excel file spreadsheet has now been included as supplemental data which contains all of the numerical data underlying all figures.
